# Brain ventricle volume correlates with effortful control in healthy young males

**DOI:** 10.1186/1471-2202-13-S1-P22

**Published:** 2012-07-16

**Authors:** Rongxiang Tang, Yi-Yuan Tang

**Affiliations:** 1 South Eugene High School, Eugene, OR 97401, USA; 2 Department of Psychology, University of Oregon, Eugene, OR 97401, USA; 3 Texas Tech Neuroimaging Institute and Dept of Psychology, Texas Tech University, Lubbock, TX 79409, USA

## 

The neuroanatomical correlates of temperament in human have been examined extensively but not comprehensively. Most studies have focused on certain brain regions of gray and white matter, such as prefrontal cortex, amygdala, anterior cingulate cortex, etc, however, less is known about the contribution of other areas to human temperament [1-4]. Previous studies have shown that brain ventricle is related to psychiatric diseases, which exemplify problems in self-control, here we sought to explore whether the size of brain ventricle is associated with individual differences in temperament in healthy college students.

Thirty-eight subjects (21 males, 17 females, age 18-23 years) were selected randomly, and scanned using a three-dimensional T1-weighted magnetization-prepared gradient echo (MPRAGE) sequence on a Siemens 3T Allegra scanner to acquire anatomic data, which were then segmented by FreeSufer and calculated for volume. Individual temperament was assessed using the Adult Temperament Questionnaire (ATQ)[5], and scores of each scale were then correlated with the ventricular volume.

We found a positive correlation between the volume of fourth ventricle and effortful control (one of the four core temperament dimensions in ATQ) in males (r=0.485, p=0.026), suggesting that males with larger fourth ventricle volume, have great capacity to focus and direct attention, and inhibit inappropriate behavior. However, no significant correlation was detected in females.

## Conclusions

Our results indicated that the fourth ventricle, a new region of interest in the brain other than the widely accepted anterior cingulate cortex, is also related to aspects of effortful control in human. Additionally, our results also supported finding from previous studies, that is, the biological mechanisms of temperament differ between sexes.
